# Endoscopic scissors for removal of plastic tubes trapped with silk lines

**DOI:** 10.1055/a-1810-6911

**Published:** 2022-04-27

**Authors:** Zhihui Duan, Shengyun Zhou

**Affiliations:** Digestive Endoscopy Center, Xingtai People’s Hospital, Xingtai, China

Endoscopic scissors have the advantage of preventing potential complications associated with thermal and mechanical damage to surrounding structures 1 . They can be used on insulated cores, metallic coils, fish bones, and nasogastric tubes 1 2 3 . However, there have been no reports of endoscopic scissors being used to cut through silk line.

**Fig. 1:**
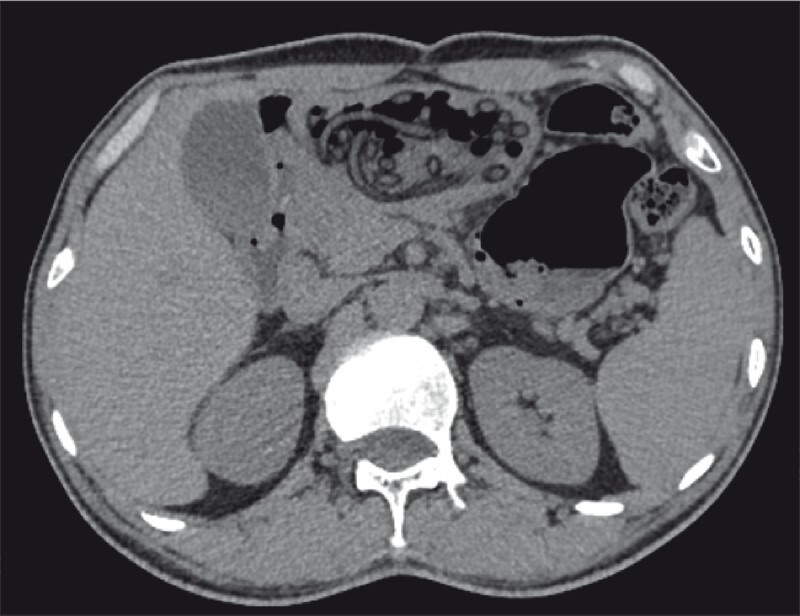
Multiple tubular objects in the stomach and duodenum on the computed tomography scan.

**Fig. 2:**
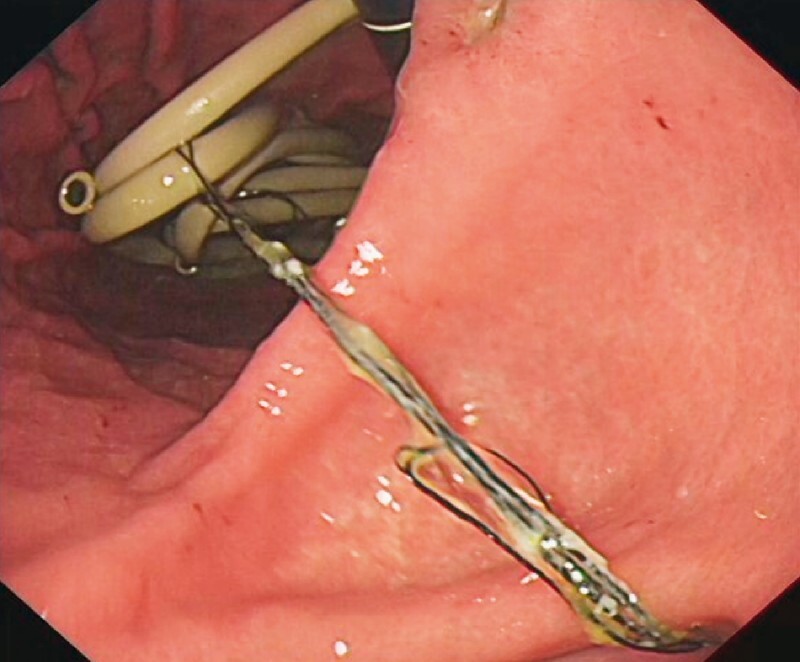
Numerous yellow tubes in the stomach. One end of the silk line was tightly wound around the tubes in the stomach.

**Fig. 3:**
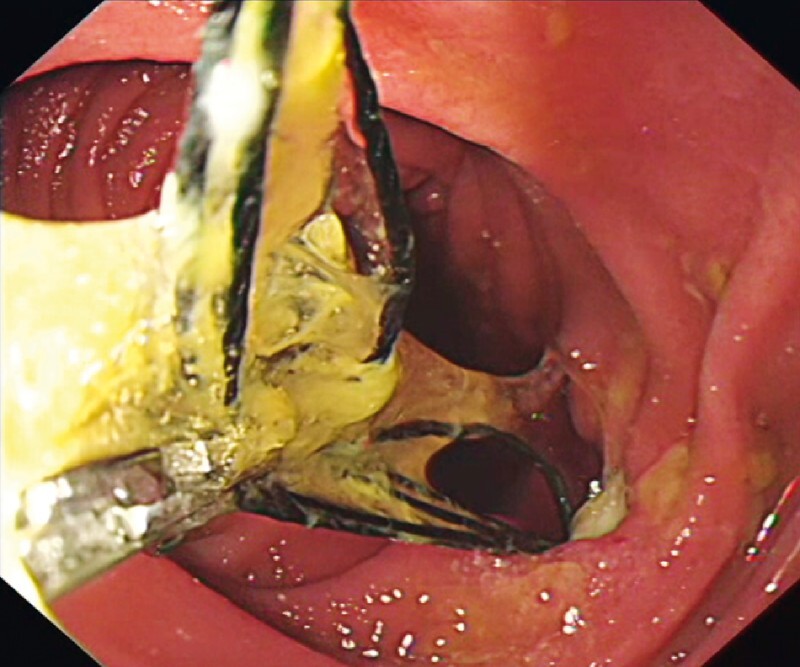
Numerous yellow tubes in the duodenum. One end of the silk line was embedded in the duodenal ulceration. Many extraction attempts were made, but the silk line remained in the duodenum.

**Fig. 4:**
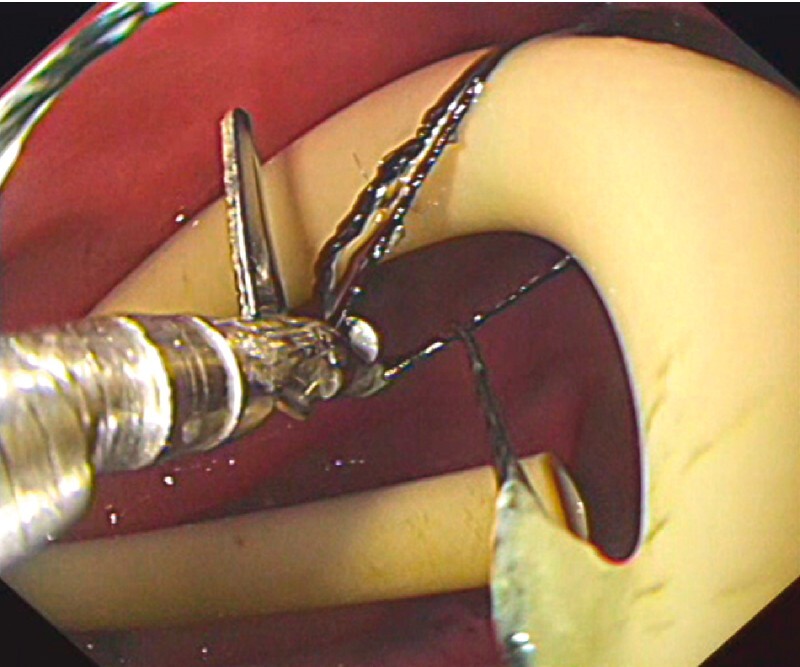
Endoscopic scissors were used to successfully cut through the silk line.

**Fig. 5:**
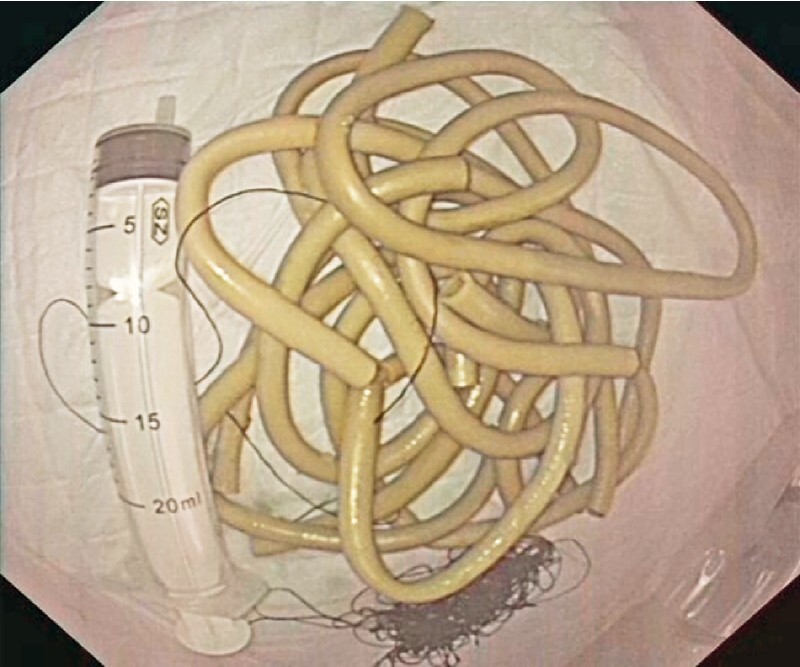
All identified foreign bodies (yellow plastic tubes and silk lines) were removed. A 20-ml syringe is pictured as a reference.

A middle-aged man presented to the emergency department with abdominal pain for the past eight days. He disclosed that he had ingested a long plastic tube trapped with a silk line after drinking two years ago. Physical examination was normal, as was his routine blood test. Computed tomography scans showed multiple tubular objects in the stomach and duodenum ( Fig. 1 ). Gastroscopy revealed numerous yellow tubes in the stomach and duodenum ( Fig. 2 , Video 1 ). It was observed that one end of the silk line was wedged in the duodenum wall with secondary mucosal ulceration ( Fig. 3 ), and the other end was tightly wound around the tubes in the stomach ( Fig. 2 ). Because the silk line was very flexible, the endoscopy nurse made several attempts before successfully cutting it in half while pulling silk line tightly ( Fig. 4 ). All identified foreign bodies were removed with a rat-tooth forceps ( Fig. 5 ). The patient recovered well and was discharged 8 days after the procedure.

**Video 1** Endoscopic scissors for removal of plastic tubes trapped with silk lines.


We highlight the role of endoscopic scissors in cutting through silk line in this challenging case, which prevented further surgical removal of the foreign bodies. Endoscopic scissors, originally designed for cutting the nasobiliary duct in vivo 2 , were adopted to cut the silk line in half in our case. As far as we know, this is the first case of plastic tubes trapped with silk lines to be removed using endoscopic scissors.

Endoscopy_UCTN_Code_TTT_1AO_2AL
